# Supplementary material for: MetaRibo-Seq measures translation in microbiomes
Source: Nat Commun. 2020 Jun 29;11:3268. doi: 10.1038/s41467-020-17081-z (PMC7324362; doi:10.1038/s41467-020-17081-z)
Supplement: Supplementary file 10 — Supplementary Data 7 [file 41467_2020_17081_MOESM10_ESM.zip › File2/Confidence_High_Taxonomy/431597_out.krona.html]

Javascript must be enabled to view this page.

members
magnitude
magnitudeUnassigned
count
unassigned
taxon
rank

431597\_out

15

15
2
superkingdom

phylum
201174
15

15
1760
class

15
order
85004

family
31953
15

1678
genus
15

species
28026

SRS097920\_contig\_number\_23385SRS098073\_contig\_number\_15063
2

species
1680

SRS049446\_contig\_number\_12753SRS050752\_contig\_number\_22433SRS056519\_contig\_number\_17683SRS098717\_contig\_number\_23500SRS1055022\_contig\_number\_22693SRS1055056\_contig\_number\_12981SRS142890\_contig\_number\_contig-100\_1209.240007SRS142890\_contig\_number\_contig-100\_1801.240599SRS143148\_contig\_number\_25603SRS144183\_contig\_number\_11562SRS145497\_contig\_number\_contig-100\_14.122213SRS147919\_contig\_number\_18637SRS149325\_contig\_number\_contig-100\_17.99467
13
